# Health information-seeking behavior among users of traditional, complementary and integrative medicine (TCIM)

**DOI:** 10.1186/s12906-025-04843-9

**Published:** 2025-03-21

**Authors:** Miriam Trübner, Alexander Patzina, Judith Lehmann, Benno Brinkhaus, Christian S. Kessler, Rasmus Hoffmann

**Affiliations:** 1 Otto-Friedrich-University Bamberg, Bamberg, Germany; 2 Johannes Gutenberg University Mainz, Mainz, Germany; 3 Institute for Employment Research, Nuremberg, Germany; 4 Institute of Social Medicine, Epidemiology and Health Economics, Charité - Universitätsmedizin Berlin, corporate member of Freie Universität Berlin and Humboldt-Universität zu Berlin, Berlin, Germany; 5 Department of Internal Medicine and Nature-Based Therapies, Immanuel Hospital Berlin, Berlin, Germany

**Keywords:** Traditional medicine, Complementary medicine, Integrative medicine, Alternative medicine, Medical advice, Medical information, Health information-seeking behavior

## Abstract

**Background:**

The use of traditional, complementary, and integrative medicine (TCIM) is widespread among the German population and driven by various motives, including both supplementing and avoiding treatments with conventional medicine. The aim of this article is to examine how these motives relate to different health information-seeking behaviors.

**Methods:**

The study uses regression analysis based on data from a German online access panel, which explored the use and acceptance of TCIM in Germany in 2022. From this study, we use information on 1,696 individuals (aged 18–75 years) who vary in their motives for using TCIM (subjective statements on five-point Likert scales) and have used TCIM to treat health problems.

**Results:**

Overall, TCIM is considered more a health-promoting measure than it is driven by aversion towards conventional medicine. Our analysis of information-seeking behavior for certain therapeutic procedures reveals that, as respondents’ propensity to use TCIM as a health-promoting measure rises, they are more likely to perceive themselves as being influenced by scientific studies (AME: 0.04, *p* = 0.004), personal advice (AME: 0.09, *p* = 0.000), and their social circle’s experiences (AME: 0.08, *p* = 0.000). In contrast, respondents who use TCIM more due to aversion to conventional medicine are less likely to perceive themselves as being influenced by scientific studies (AME: -0.04, *p* = 0.004) and doctors (AME: -0.07, *p* = 0.000). When analyzing respondents’ most important medical information source, our results reveal that the more individuals indicate using TCIM out of aversion, the more likely they are to consider (online) media outlets their most important medical resource (AME: 0.05, *p* = 0.000), while the likelihood of considering medical professionals most important decreases (AME -0.06, *p* = 0.000).

**Conclusion:**

Motives behind TCIM use vary and correspond to differences in individuals’ health information-seeking behavior. Beyond these motive-related differences, TCIM users value sources of health information other than their medical practitioners. This calls for an intensification of TCIM training among medical professionals to provide high-quality consultation and the creation of reputable online portals to ensure the provision of trustworthy information about TCIM.

**Supplementary Information:**

The online version contains supplementary material available at 10.1186/s12906-025-04843-9.

## Background

In recent decades, Traditional, Complementary and Integrative Medicine has gained significant prominence among Western populations [1–3]. TCIM is an umbrella term synonymous with TCIH: Traditional, Complementary, and Integrative Healthcare [4] (also used by the WHO [5]) to refer to a wide range of therapeutic practices applied alone or in combination with conventional medicine. Until recently, the term “Complementary and Alternative Medicine” (CAM) was commonly used, especially in the Anglo-American context [6], but this has now been expanded to better reflect the diversity of medical approaches on a global scale. In this context, it should be noted that specifically for Germany, TCIM also encompasses “Traditional European Medicine”, known in Germany as *Naturheilkunde*. For more information on the development of the terminology see Jeitler et al. [7].

These terminological developments mean that existing research mainly discusses the use and acceptance of CAM, but it can largely be transferred to what we (analogously to WHO terminology) define as TCIM. To be consistent, we refer in the following to TCIM even though most cited authors use the acronym CAM in their research papers. In particular, a substantial body of research identifying sociodemographic and health-related factors related to the use of TCIM has established that women [2, 8–13], middle aged individuals [2, 8, 10, 14], and individuals with higher levels of education and income [1, 2, 8–13], poor health [8, 9, 15], and chronic diseases [8] are more likely to use TCIM. Further, studies also show that people with high levels of spirituality are more likely to use and favor TCIM [16, 17].

Besides these individual characteristics, research indicates three different or interrelated motives behind TCIM use: First, the main motive for, and application of, TCIM is the combination of TCIM with conventional medicine as a supportive health measure. In this context, most individuals use TCIM in combination with conventional medicine for minor ailments [18] or use TCIM complementarily to conventional medicine for severe illnesses and treatments such as cancer, frequently to reduce the side effects of conventional treatments [19]. Second, studies also show a link between TCIM use and a desire to promote overall well-being [20, 21] irrespective of ailments. Third, a less common but identified motive in the literature is an aversion towards conventional medicine as a motive for the use of TCIM to avoid conventional medicine. Results of a study by Siapush [22] specifically emphasize negative doctor–patient experiences leading individuals to favor TCIM. Other studies show that people holding both general conspiracy beliefs [23] and medical conspiracy beliefs [24] are more likely to lean towards TCIM as an alternative to conventional medicine. Also individuals with unmet medical needs are more likely to use TCIM [2].

In summary, transferred to the application of TCIM, two main motives of use can be identified for the treatment of illnesses: First, TCIM is used in addition to or in combination with conventional medicine as a supportive health measure; second, TCIM is chosen because of individuals’ aversion to conventional medicine, and used as a substitute for conventional medical treatments. In this study, we determine the empirical relevance of these two potential motives for using TCIM. Additionally, TCIM is also used to promote overall well-being through self-initiative, irrespective of health issues; this is not the focus of this study. Consequently, the motives behind the use of TCIM are connected to different fields of TCIM application, which may be partly unrelated to common treatments prescribed by medical practitioners. Furthermore, this study examines where individuals using TCIM get their medical information from, what they assess as the most important source of medical information, and how they use different sources for therapeutic decision making. Understanding the health information-seeking behavior of users of TCIM is important to help identify which measures are needed to assist the appropriate application of TCIM.

With the advances of digitalization, medical information in general and information on TCIM in particular is not limited to medical professionals as a source, but is nowadays also easily available to laypersons through publicly available sources, both online and offline, such as web searches, blogs, apps, newspapers, and books. A Eurostat survey among 16- to 74-year-olds on the use of information and communication technologies in households revealed that, in 2019 in Europe, 55% percent of respondents reported having looked for health information online within the last three months. In Germany, the share of people searching online for medical information is as high as 70% [25]. Hence, while access to medical information has become more democratic overall [26], we also know that some subpopulations are more likely to use the internet to seek medical information, such as women and younger people [27, 28], or people with chronic illnesses or poorer health [29]. This development enables people to actively engage in healthcare-related decisions and to proactively obtain health information, especially regarding information on treatments in the field of TCIM, which are not part of (or, if they are, are only one subordinate aspect of) mainstream conventional medical treatments in Germany.

However, despite the spread of and access to various sources for medical information, studies show that medical advice by doctors is still favored, and online and offline sources are likely to be used in combination [30]. Asked about their rationale for searching the internet for medical advice before visiting a general practitioner, patients report their desire for a balanced doctor–patient relationship and self-care, as well as emphasizing their efforts to best prepare for the typically brief medical consultations [31]. In this context, trust between patient and doctor is paramount not only on the part of the patients, who initiate and maintain the interaction with the doctor, but also on the part of the medical professional to ensure that patients follow the medical instructions that are crucial to their recovery [32, 33]. The healthcare system, with its standards and regulations, is principally responsible for laying the groundwork for trust in healthcare professionals [32]. In contrast, social media use is often linked to individuals seeking social support and interactions with others [34], something which is not available, or only to a limited extent, in patient–doctor interactions.

It is important to note that laypersons also obtain information from their personal social circle, such as family members, friends, co-workers, or other acquaintances, influencing people’s health decisions [35]. Other more traditional sources for medical information are books, magazines, leaflets, TV or the radio, characterized, however, by a one-way flow of information [36]. Studies have found that health-conscious individuals committed to healthy behaviors rely on interpersonal information sources, while passive sources like TV and radio are more commonly used by those who are less focused on health [37].

While research has primarily focused on the influence of mass media on individuals’ health behavior [38], this study shifts the focus to investigating how the relevance of the different motives for the use of TCIM relate to individuals’ health information-seeking behavior, irrespective of their demographic characteristics. Therefore, our main question is how individuals perceive the influence of various information sources once the preference for medical approaches is established.

Based on a novel survey that provides detailed insights into the current use and acceptance of TCIM within the German population, our study is to our knowledge the first to analyze the health information-seeking behavior of TCIM users, and as such offers valuable implications for health policy measures.

### Theoretical framework and hypothesis

The Health Belief Model (HBM) [39, 40] provides a useful theoretical framework for understanding how diverse TCIM users perceive the influence of different information sources. Initially developed to explain preventive health behavior change, HBM has been adopted by health communication research to explain how information is processed [41]. The HBM suggests that individuals are more likely to take preventive action against illness if they believe they are at risk of a disease (*susceptibility*) and/or if they think the disease could have serious consequences (*severity*). Further, they are motivated if they believe that a specific action could reduce the threat of a disease (*benefits*), and if they perceive few obstacles to taking action (*barriers*). Individuals are more likely to engage in preventive health behavior if they believe they can achieve the desired outcome (*self-efficacy*). Lastly, *cues of action* are the factors in individuals’ surroundings and social circles that can impact behavior change.

Transferred to the health information-seeking behavior of TCIM users, the *susceptibility* to and *severity* of a health problem is mainly a factor influencing the actual motive for using TCIM, not necessarily influencing the information-seeking behavior once the decision is made to use TCIM (be it out of aversion towards conventional medicine or from a health-enhancing perspective). However, the HBM is directly relevant for other aspects of health information-seeking behavior: One the one hand, nonmedical information sources make it considerably easier to access specific TCIM information, posing minimal *barriers* to obtaining it: This accessibility and ease of use may also help promote *self-efficacy* in health behavior and treatment regardless of the motive behind TCIM use. On the other hand, medical information sources could also be valuable to TCIM users by offering guidance on how to safely integrate TCIM into their overall treatment plan, depending on the extent to which users trust their doctor’s advice; this will increase the *benefits* of these information sources. TCIM users who show an aversion towards conventional medicine will, conversely, find medical information less beneficial. Consequently, this variety in the benefits and barriers an individual TCIM user ascribes to different sources of health information – that is, to at least some of their *cues of action* – will differ according to their motives for using TCIM. Taking into account the diverse motives of using TCIM, we hypothesize the following:

#### H1a

The higher the aversion towards conventional medicine as a motive for using TCIM, the more likely TCIM users are to consider nonmedical information sources to be their most important source of information (they will ascribe greater benefits to nonmedical information sources; perceive fewer barriers to informing themselves about it; and feel a greater sense of self-efficacy when using nonmedical information sources).

#### H1b

Similarly, the higher the aversion towards conventional medicine as a motive to use TCIM, the more likely TCIM users are to perceive nonmedical information regarding therapeutic advice as influential overall (again, these users will ascribe greater perceived benefits to nonmedical information sources, and exhibit greater levels of self-efficacy because of their sense of obtaining their health information independently).

#### H2a

Conversely, the more TCIM users use TCIM as a health-promoting measure in combination with conventional medicine, the more likely they are to consider medical advice as the most important source of health information (these users will rate the perceived benefits of professional medical information highly, and perceive fewer barriers to obtaining and applying it).

#### H2b

The more TCIM users use TCIM as a health-promoting measure in combination with conventional medicine, the more likely TCIM users are to perceive a variety of information sources for therapeutic advice as influential (perceiving a well-rounded sense of the benefits of various TCIM information sources, and a high level of self-efficacy due to their diverse health information-seeking behavior).

## Methods

### Data and sample

This cross-sectional study is based on a survey on the “Use and Acceptance of TCIM in Germany”, which was conducted in fall 2022 with an online access panel with quota sampling, in collaboration with the German research institutes Sinus, Respondi, and Conversio. The survey covers a wide range of aspects of TCIM, encompassing knowledge, experiences, attitudes, and the application of these approaches, but also questions on attitudes towards conventional medicine. Related topics and factors include general attitudes, current health status, medical history, and basic sociodemographic information. Further questions in the survey covered nutrition, Covid vaccination, Ayurveda, the Sinus milieu indicator, and the EQ-5D-5L quality of life questionnaire (see [7] for an overview of the study).

The study was approved by the Charité University Ethics Committee and registered with ClinicalTrails.gov (NCT05530720), and participants gave informed consent before taking part. In total, 8,821 out of 41,011 invited panelists participated in the survey, giving an overall response rate of 21.5%. Out of the 8,821 respondents who began the survey, 453 cases (5.1%) were excluded based on criteria such as lack of consent and age. Additionally, 2,845 respondents (32.3%) were omitted because of filled quotas, and 313 (3.5%) removed due to failed internal quality checks. 1,000 (11.3%) individuals did not complete the survey. Since the quota sampling was effective only for the age group of 18–75-year-olds, the final sample included 4,065 respondents. As we are only interested in respondents who have already used TCIM for certain diseases (44.0%) (acute or chronic respiratory diseases, acute or chronic gastrointestinal diseases, allergies, diabetes mellitus, skin diseases, cardiovascular diseases, childhood diseases, headache disorders, cancer, neurological diseases, mental illness, thyroid disorders, pain disorders of the musculoskeletal system), the sample used in this part of the study is reduced to 1,788 respondents. Finally, we dropped 17 cases where we had missing information for the dependent variable on the respondents’ most important medical source, and another 75 cases with too many missing responses for the seven items on motives for the use of TCIM (see measures section for details), leaving us with a final sample of 1,696 respondents (see supplementary material 2: Table A1 for descriptives of the sample and the variables used for this study).

### Measures

#### Dependent variables

Respondents were asked to state which of the following sources is most important to them for medical information: active search on the internet (e.g. Google) (1), social media (e.g. Instagram, Facebook, TikTok, messengers) (2), TV and radio (3), magazines and newspapers (4), medical professionals (e.g. doctors, alternative practitioners) (5), family (6), friends or acquaintances (7), others (open question) (8). Out of 26 answers in the open category ‘others’, nine answers could be assigned to one of the preceding categories; the other 17 cases were excluded from the analyses. Out of these seven answer categories, we generated the variable *most important medical source* with three outcomes: *medical professionals* (answer category 5), *(online) media outlets* (combining answer categories 1 to 4), and *social circle* (combining answer categories 6 and 7).

Respondents were also asked to state the extent to which the following aspects influence their decision-making regarding therapeutic procedures: results of scientific studies (1), medical recommendation by doctors (2), personal recommendation (3), experiences of family, friends, and acquaintances (4). Answer categories range on a five-point scale from ‘does not influence my decision’ (1) to ‘influences my decision enormously or very strongly’ (5). We dichotomized answer categories into ‘does not influence’ (0), combining answer categories 1 to 3, and ‘does influence’ (1), combining answer categories 4 and 5.

#### Independent variables

*Motives for TCIM use*: Respondents were asked to assess on a 5-point scale (from (1) ‘completely unimportant’ (1) to ‘very important’ (5)) the relevance of the following reasons for using Traditional European Medicine (*Naturheilkunde*), complementary medicine, integrative medicine, or alternative medicine, thus covering the main areas of application of TCIM identified in former studies: (1) ‘fewer side effects than with conventional medicine’, (2) ‘reduction of side effects of conventional medication’, (3) ‘better chances of recovery’, (4) ‘improving my health literacy and self-care competencies’, (5) ‘I do it out of health-related desperation’, (6) ‘I don’t like conventional medicine’, (7) ‘I have had bad experiences with conventional medicine’. We calculated two indices: *health-promoting measure*, based on the mean value of the items 1 to 4, and *aversion to conventional medicine*, based on the mean value of the items 5, 6 and 7. We dropped cases where either no item for the first index or no item for the second index was given (*N* = 75).

An alternative way of reducing dimensionality, and often preferred to the use of indices, is via Principal Component Analysis (PCA). When calculating an index by the mean of a certain set of variables, every item used for the index has the same weight. In a PCA, items are weighted differently, allowing items to contribute to the dimension with different weights, enabling the extraction of more precise dimensions. Moreover, PCA *exploratively* extracts the dimension based on the variability and correlation of the items. The PCA for this data confirms our assignment of the items to these two dimensions (for details on the PCA solution see Supplementary material 1). However, as PCA only considers cases with complete information on the motives for TCIM use, using PCA for the reduction of the dimensionality of these items would have resulted in an exclusion of 335 cases (~ 20%). To avoid this major loss of cases, we used the indices for the subsequent multivariate analysis instead.

#### Confounders

The following confounders are assumed to be related to the motives for TCIM use and the sources of information perceived as valuable. First, we controlled for the initial sources of influence for using TCIM, including: ‘family/friends have had good experiences’, ‘advice from my treating physician’, ‘I heard about it in the media’. Respondents were asked to assess how influential they considered these sources on a 5-point scale (from (1) ‘completely unimportant’ (1) to ‘very important’ (5)). We recoded these answers as dummy variables, with information sources ultimately classified as either important (1) or not (0).

Next, we controlled for sociodemographic variables. Resources are operationalized by *level of education* (comprising ‘low’, ‘medium’, ‘high’, and ‘currently enrolled’) and *net equivalent household income*^1^. Individual factors include *gender* (‘men’, ‘women’, ‘diverse’), *age*, and *work status* (‘full time’, ‘part time’, ‘in training/school’, ‘not working’). As a contextual factor we included *hometown size* (‘under 2,000 inhabitants’, ‘2,000 to under 5,000 inhabitants’, ‘5,000 to under 20,000 inhabitants’, ‘20,000 to under 50,000 inhabitants’, ‘50,000 to under 100,000 inhabitants’, ‘100,000 to under 500,000 inhabitants’, ‘500,000 inhabitants and more’)

For health-related factors, we control for the *subjective health status* ranging from ‘very bad’ (1) to ‘excellent’ (5), and whether respondents have any *existing medical training* (differentiating between ‘no medical training’ (0), ‘medical training’ (1)).

Further, we use the level of *spirituality* as a confounder, including the responses ‘yes, very’ (1), ‘yes, somewhat’ (2), ‘neither nor’ (3), ‘not really’ (4), ‘not at all’ (5), and ‘don’t know’ (6). Finally, we control for the *importance of digital device*s in respondents’ daily lives. The answer categories here comprise ‘does not apply’ (1), ‘does not really apply’ (2), ‘neutral’ (3), ‘applies somewhat’ (4), ‘fully applies’ (5), and ‘don’t know’ (6). For the items on spirituality and digitality the ‘don’t know’ and ‘neither nor’ categories are combined to be able to include the variable in the model as a metric variable.

### Analytical strategy

First, we apply multinomial logistic regression models to reveal the association between the importance of the motives for TCIM use and the ternary outcome variable ‘most important source of medical information’ with the values *medical professionals*, *(online) media outlets*, and *social circle*. Second, based on separate logistic regression models, we further examine how motives for TCIM use are related to the perceived importance of various sources (*scientific studies*; *medical recommendation by doctor*; *personal recommendation*; *experiences of family*,* friends*,* and acquaintances*) when taking therapeutic decisions. To interpret the regression coefficients, we computed average marginal effects (AME), the average effect of any independent metric variable x_i_ on the probability of the dependent variable y being 1, and average discrete changes (ADC), the average effect of any independent categorical variable x_i_. In doing so, we can identify the probability of the dependent variable being 1 when the independent variable increases by 1 scale point and compare stepwise logistic regression models [42]. To avoid confounding bias and to approximate the causal effect as closely as possible with the cross-sectional data at hand, we conditioned on gender, age, hometown size, education, net equivalent household income, work status, medical training, subjective health status, level of spirituality, and role of digitality in our analyses.

## Results

### Descriptives

**Fig. 1:**
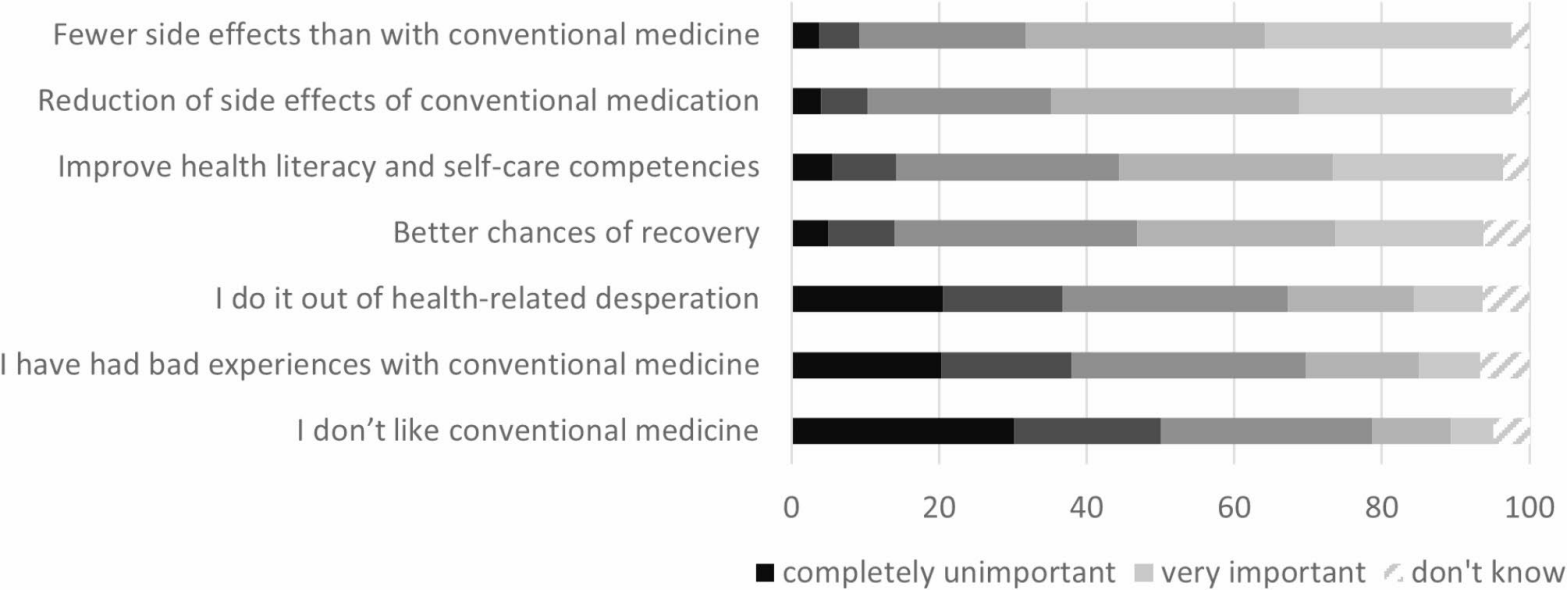
Evaluation of motives for using TCIM (*N* = 1,696). Note: Authors’ own calculations, results unweighted

As illustrated in Fig. 1, the main motive for using TCIM is seen in the desire for fewer side effects compared to conventional medicine, followed by the reduction of the side effects of conventional medication, improving one’s health literacy and competencies, and hopes of improving their chances of recovery when sick. The items rated with the least relevance are health-related desperation, bad experiences, and aversion towards conventional medicine.

As described in the method section, we combined these seven items into two indices. The first index, *health-promoting measure*, is based on the average score of items 1 to 4; the second index, *aversion towards conventional medicine*, is based on the average score of items 5 to 7. Overall, using TCIM as a health-promoting measure is on average rated as ‘important’ (overall mean: 3.7; SD: 0.9), and TCIM use motivated by an aversion towards conventional medicine is rated as neither ‘very important’ nor ‘completely unimportant’ (overall mean: 2.6; SD: 1.0), demonstrating that the main justification for TCIM is the desire to promote overall health.

### Motives for TCIM use and health information-seeking behavior

Table 1 displays the AMEs and ADCs of the multinomial regression model (full results with displayed confounders are in the supplementary material 2: Table A2). The relevance of using TCIM as a health-promoting measure is unrelated to the preference for different sources of medical information (M1a). This finding holds even after controlling for confounding variables (M1b), thus refuting H2a, where we expected that this motive for the use of TCIM would be associated with perceiving doctors as the most important source of influence. In contrast, a higher level of aversion towards conventional medicine as a motive for using TCIM is positively associated with viewing media outlets as the most important medical source and negatively associated with viewing medical professionals as the most important medical source (M1a). Coefficient sizes drop only marginally after controlling for confounding variables (M1b). These results represent empirical evidence for H1a, where we expected that aversion towards conventional medicine would be associated with perceiving nonmedical sources as most important.

**Table 1:** Results of multinomial regression of most important source of medical information on motives for TCIM use

	M1a			M1b		
	medical profess.	(online) media outlets	social circle	medical profess.	(online) media outlets	social circle
	AME (Std. err.)	AME (Std. err.)	AME (Std. err.)	AME (Std. err.)	AME (Std. err.)	AME (Std. err.)
health-promoting measure	0.00 (0.01)	0.00 (0.01)	-0.00 (0.01)	-0.01 (0.02)	-0.02 (0.01)	-0.01 (0.01)
aversion towards conventional medicine	-0.08^***^ (0.01)	0.07^***^ (0.01)	0.01* (0.01)	-0.06^***^ (0.01)	0.05^***^ (0.01)	0.01 (0.01)
confounders	no	no	no	yes	yes	yes
N	1,696			1,696		

Note: Significance level: * *p* < 0.05, ** *p* < 0.01, *** *p* < 0.001; authors’ own calculations

**Fig. 2:**
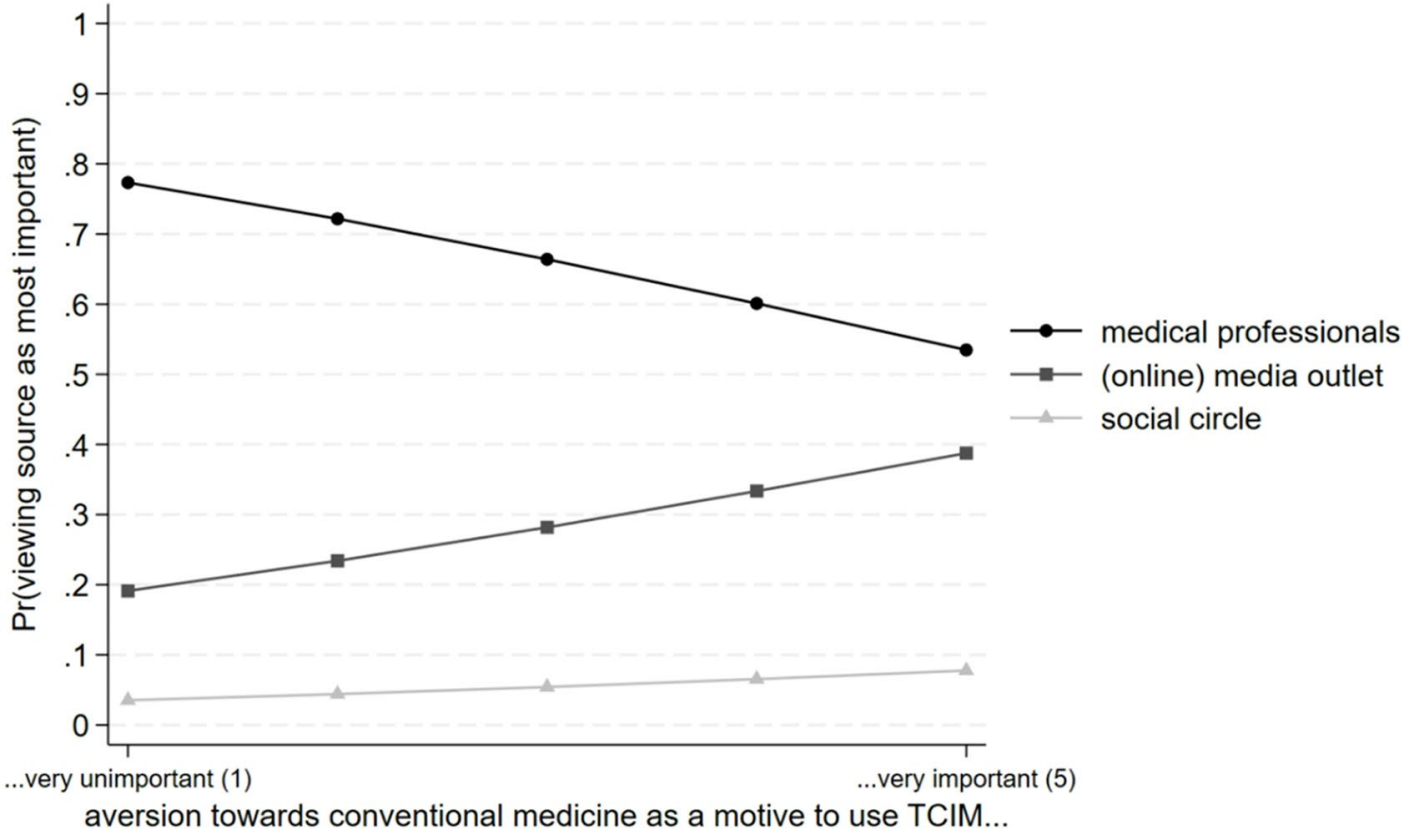
Predicted probabilities for the motive of aversion towards conventional medicine and main source of medical information (*N* = 1,696)

Figure 2 illustrates the AMEs for the level of aversion towards conventional medicine as a motive for TCIM use and the corresponding predicted probabilities for viewing one of the three sources for medical information as valuable. The predicted probability for viewing advice by medical professionals as most important is just under 80% when aversion towards conventional medicine is not relevant for TCIM use.

When aversion towards conventional medicine as a reason to use TCIM was very important, only slightly more than 50% of respondents view medical professionals as the most important source of medical information. Correspondingly, about 40% view (online) media outlets as the most important source when seeking health information.

Next, we analyze how respondents view the influence of various sources on their decision for therapeutic approaches dependent on the relevance of the two motives for TCIM use. Table 2 displays the AMEs and ADCs of the logistic regression model (full results can be found in the supplementary material 2: Table A3). We establish that, with increasing relevance of TCIM usage as a health-promoting measure, there is a statistically significant increase in the likelihood of users perceiving themselves as being influenced by the medical advice of doctors or scientific studies regarding therapeutic procedures (M2a and M2b (not for doctors); M3a and M3b). The same association, with stronger coefficient size, is found for the perceived influence of personal advice and the experiences of the social circle (M4a and M4b; M5a and M5b). All models provide empirical evidence in favor of H2b, where we expected that viewing TCIM as a health-promoting measure is associated with perceiving both medical and nonmedical sources of information as influential.

In contrast, the level of aversion towards conventional medicine as a motive for TCIM use is negatively associated with scientific studies and medical doctors as a perceived source of influence. This provides empirical evidence in favor of H1b, where we expected that aversion towards conventional medicine as a motive for using TCIM is associated with not perceiving medical information sources as influential.

**Table 2:** Results of logit regressions of importance of sources for taking therapeutic decisions on motives for TCIM use

	M2a	M2b	M3a	M3b	M4a	M4b	M5a		M5b
	scientific studies		advice by doctor		personal advice		experience of social circle		
	AME (Std. err.)		AME (Std. err.)		AME (Std. err.)		AME (Std. err.)		
health-promot. measure	0.08^***^ (0.01)	0.04^**^ (0.01)	0.05^***^ (0.01)	0.00 (0.01)	0.16^***^ (0.01)	0.09^***^ (0.01)	0.15^***^ (0.01)		0.08^***^ (0.01)
aversion to conv. med.	-0.04^**^ (0.01)	-0.04^**^ (0.01)	-0.10^***^ (0.01)	-0.07^***^ (0.01)	0.03^*^ (0.01)	0.02 (0.01)	0.01 (0.01)		0.01 (0.01)
confounders	no	yes	no	yes	no	yes	no		yes
N	1,696	1,696	1,696	1,696	1,696	1,696	1,696	1689*	

Note: Significance level: * *p* < 0.05, ** *p* < 0.01, *** *p* < 0.001. In Model 5b seven observations were omitted as there is no variation in the dependent variable for people currently enrolled in education. Authors’ own calculations

As a robustness check, we also calculated the multivariate analysis with the scores retrieved by PCA (based on the smaller sample), which yields essentially the same results (for multivariate results based on the PCA see Supplementary material 1).

## Discussion

The purpose of the analysis of this cross-sectional study was the identification of health information-seeking behavior of TCIM users in Germany dependent on the relevance they assign to their motives for using TCIM. Differentiating between the relevance of using TCIM as a health-promoting measure and using it out of aversion towards conventional medicine, we establish that, depending on the relevance respondents ascribe to either motive for TCIM use, different information sources are valued and consulted irrespective of the initial sources influencing individuals’ motive for using TCIM, and of sociodemographic and health-related background information. Overall, we observe that ascribing relevance to TCIM as a health-promoting measure was not associated with favoring one information source over the other, and was associated with an openness to professional and non-professional advice when making medical decisions; this openness to various sources of information may be explained by the perception of the lower barriers associated with obtaining information from these sources, improving perceived self-efficacy. One implication of this finding is that general practitioners should be advised to ask patients about non-prescription use of TCIM and supplements, to better disclose the pros and cons of these treatments. This necessitates, on the one hand, that doctors and other healthcare providers receive more training regarding TCIM, and, on the other hand, balanced, high-quality doctor–patient communication (this has in any case been proven to be essential for patients to establish trust in their doctors) [43]. Accordingly, as patients’ trust grows thanks to their doctors’ willingness to discuss TCIM with them, the patients’ willingness to share information on their TCIM-related self-medication and treatment increases concomitantly, which studies have shown is often hidden from doctors [44].

Aversion towards conventional medicine was, on average, rated less relevant in motivating TCIM use than the desire to use TCIM as a health-promoting measure. Looking at the relationship between the relevance of this aversive motive and health information-seeking behavior, we find that high levels of aversion towards conventional medicine increase the probability of being reluctant to take advice from others in general, but especially from doctors; one potential theoretical mechanism here may be that, for these users, the perceived benefits of medical information sources are low. Notably, respondents were considerably less likely to state that doctors were their most important source of medical information when their level of aversion towards conventional medicine is high. In this case, individuals are more likely to attempt to reduce the perceived barriers to obtaining health information by using universally accessible (online) media outlets, also preferring to maximize their perceived self-efficacy by making health-related decisions autonomously.

This finding – that (online) media outlets can be ascribed greater informational value than professional medical advice – raises critical questions, as individuals searching online (especially if this approach is their only source of health information) might be affected by confirmation bias [45, 46]. In this regard, studies on online health information-seeking behavior show that people not only choose sources which align to their present attitudes on the subject [45, 46], but that they also evaluate these sources as more credible and useful [45], and that biased online searches can contribute to false beliefs [47]. Based on these findings, and the popularity of TCIM, it is important that future research investigates potential confirmation bias in the TCIM-related online health information-seeking behavior for those TCIM users with an aversion to advice from medical professionals.

Moreover, independent search behavior for medical advice may lead to self-treatment and self-medication; this may harbor risks where so-called ‘natural’ treatments are used, but also for conventional self-treatments, as they may cause side effects when overdosed or used with other medication, or may lead to the delayed introduction of potentially effective conventional medicine. In this context, pharmacies or other health care providers with a face-to-face consultancy play a relevant role in providing information to people buying non-prescription TCIM products, in pointing out potential side effects, and in informing customers when medical products are not evidence-based. This (additional) professional advice is lacking when TCIM products are purchased online or when TCIM treatments encountered via (online) media are applied unsupervised. Therefore, reputable online portals need to be established (e.g., in Germany, by the Federal Ministry of Health, medical societies from the TCIM sector, and academic consortia and faculties) to provide reliable and high-quality information about TCIM. On that note, further research needs to establish which medical products and treatments individuals use independently for which medical matters, to better evaluate the seriousness of potential health risks and benefits.

### Strength and limitations

The objective of the survey was to provide a comprehensive foundation of data that enables detailed insights into the current use and acceptance of TCIM within the German population. By doing so this study is, to our knowledge, the first to analyze the health information-seeking behavior of TCIM users, and as such offers valuable implications for health policy measures.

The survey was administered by an online access panel, which has the advantage that sensitive health information can be given without an interviewer being present. The main disadvantage is the exclusion of ‘offliners’. However, as we restricted our sample to 18–75-year-olds, the main group of offliners – i.e., the very elderly – is not part of our analytical sample. While it is possible that we generally underestimate offline health information-seeking behavior, we are interested in the association between motives and health information-seeking behavior, which is not expected to be affected by a potential underrepresentation of offline health information-seeking behavior.

One of our main variables of interest is the item about the source for medical information that is most important to respondents. While this item captures which information source is primarily used, it does not account for the relative importance of each individual source, nor does it reflect whether a combination of information sources is utilized or not. As understanding the interplay between various information sources can provide a deeper insight into the complexity of information-seeking behavior, it would be worthwhile to further investigate the relative importance of each information source. Likewise, we only have information on the respondents’ *perceived* influence of information sources for medical treatment. When interpreting the findings, it should be taken into account that the perceived influence of information sources may not fully reflect their actual impact.

Moreover, with the data at hand, we were only able to study individuals who have used TCIM for preexisting diseases or health issues. In further research, it would be valuable to also include individuals who mainly use TCIM to boost their overall health irrespective of illness. As healthy individuals consult doctors less frequently than patients with acute or chronic illnesses, studying their health information-seeking behavior might shed further light on medical practices in the important preventive phase, before health issues have developed and doctors are contacted. In this regard, a more nuanced examination of the distinct sources is also desirable, particularly with respect to the content of online search requests and websites visited.

## Conclusion

This study shows that the two motives for using TCIM are attributed different levels of relevance by respondents. On average, TCIM use as a health-promoting measure was viewed as a more relevant motivation than aversion towards conventional medicine; this distinction, in turn, corresponds to differences in health information-seeking behavior.

Those TCIM users who are motivated to use TCIM to promote their overall health do not favor one medical source over the other, as they perceive both professional and non-professional advice as beneficial regarding medical decisions. In contrast, an aversion towards conventional medicine is associated with attributing significance to (online) media outlets, which involves a potential risk due to the unassisted intake and use of medical products and treatments or the delayed introduction of potentially effective conventional medicine; the perception that barriers to TCIM information are lower may actually make it more challenging. Altogether, these findings confirm the change occurring in the contemporary healthcare system, where the doctor–patient hierarchy is becoming progressively less pronounced, and laypeople may seek to become their own health experts. Looking on the bright side, societal shifts towards patients taking more initiative and responsibility for their health will allow medical practitioners to increase the benefits of the doctor–patient interaction – especially considering the constraints of limited consultation time. This requires, however, that patients’ initiatives be taken seriously and that TCIM be integrated systematically into medical training. It also calls for the provision of reliable and high-quality online services where trustworthy information about TCIM can be found, thus contributing to the quality of medical information and the public’s understanding of health in general.

## Electronic supplementary material

Below is the link to the electronic supplementary material.

Supplementary Material 1

Supplementary Material 2

## Data Availability

Data are available upon reasonable request from the corresponding author.

## Footnotes

1 Net equivalent household income (NEI) corresponds to the individual income for individuals living alone. For households with several individuals, the NEI is based on the net household income and a score depending on the number and age of the individuals living in the household. The head of the household counts with a factor of 1.0, each additional person aged 14 and over counts with a factor 0.5, and persons under the age of 14 count with a factor of 0.3. Within the survey, net income was retrieved with 18 income categories. To approximate the NEI, we used the upper bound for the first category, the lower bound for the last category, and the medium value for all other categories.

## References

[1] Frass M, Strassl RP, Friehs H, Müllner M, Kundi M, Kaye AD. Use and acceptance of complementary and alternative medicine among the general population and medical personnel: a systematic review. Ochsner J. 2012;12:45–56. 22438782 PMC3307506

[2] Fjær EL, Landet ER, McNamara CL, Eikemo TA. The use of complementary and alternative medicine (CAM) in Europe. BMC Complement Med Ther. 2020;20:108. 10.1186/s12906-020-02903-w 32252735 10.1186/s12906-020-02903-w PMC7137515

[3] Harris PE, Cooper KL, Relton C, Thomas KJ. Prevalence of complementary and alternative medicine (CAM) use by the general population: a systematic review and update. Int J Clin Pract. 2012;66:924–39. 10.1111/j.1742-1241.2012.02945.x 22994327 10.1111/j.1742-1241.2012.02945.x

[4] von Schoen-Angerer T, Manchanda RK, Lloyd I, Wardle J, Szöke J, Benevides I, et al. Traditional, complementary and integrative healthcare: global stakeholder perspective on who’s current and future strategy. BMJ Glob Health. 2023. 10.1136/bmjgh-2023-013150 10.1136/bmjgh-2023-013150 PMC10693890 38050407

[5] World Health Organization. Traditional, Complementary and Integrative Medicine. https://www.who.int/health-topics/traditional-complementary-and-integrative-medicine#tab=tab_1. Accessed 13 Feb 2023.

[6] Brinkhaus B, Esch T, editors. Integrative medizin und gesundheit. Berlin: Medizinisch Wissenschaftliche Verlagsgesellschaft. 2021.

[7] Jeitler M, Ortiz M, Brinkhaus B, Sigl M, Hoffmann R, Trübner M, et al. Use and acceptance of traditional, complementary and integrative medicine (TCIM) in Germany – an online representative Cross-sectional study. Front Med. 2024. 10.3389/fmed.2024.1372924 10.3389/fmed.2024.1372924 PMC10965565 38545512

[8] Al-Windi A. Determinants of complementary alternative medicine (CAM) use. Complement Ther Med. 2004;12:99–111. 10.1016/j.ctim.2004.09.007 15561519 10.1016/j.ctim.2004.09.007

[9] Barnes PM, Bloom B, Nahin RL. Complementary and alternative medicine use among adults and children: United States, 2007;2008. 19361005

[10] Eisenberg DM, Davis RB, Ettner SL, Appel S, Wilkey S, van Rompay M, Kessler RC. Trends in alternative medicine use in the united States, 1990–1997: results of a follow-up National survey. JAMA. 1998;280:1569–75. 10.1001/jama.280.18.1569 9820257 10.1001/jama.280.18.1569

[11] Hunt KJ, Coelho HF, Wider B, Perry R, Hung SK, Terry R, Ernst E. Complementary and alternative medicine use in England: results from a National survey. Int J Clin Pract. 2010;64:1496–502. 10.1111/j.1742-1241.2010.02484.x 20698902 10.1111/j.1742-1241.2010.02484.x

[12] Kemppainen LM, Kemppainen TT, Reippainen JA, Salmenniemi ST, Vuolanto PH. Use of complementary and alternative medicine in Europe: Health-related and sociodemographic determinants. Scand J Public Health. 2018;46:448–55. 10.1177/1403494817733869 28975853 10.1177/1403494817733869 PMC5989251

[13] Xue CCL, Zhang AL, Lin V, Da Costa C, Story DF. Complementary and alternative medicine use in Australia: a National population-based survey. J Altern Complement Med. 2007;13:643–50. 10.1089/acm.2006.6355 17718647 10.1089/acm.2006.6355

[14] Grzywacz JG, Suerken CK, Neiberg RH, Lang W, Bell RA, Quandt SA, Arcury TA. Age, ethnicity, and use of complementary and alternative medicine in health self-management. J Health Soc Behav. 2007;48:84–98. 10.1177/002214650704800106 17476925 10.1177/002214650704800106

[15] Ong C-K, Petersen S, Bodeker GC, Stewart-Brown S. Health status of people using complementary and alternative medical practitioner services in 4 english counties. Am J Public Health. 2002;92:1653–6. 10.2105/ajph.92.10.1653 12356616 10.2105/ajph.92.10.1653 PMC1447302

[16] Ben-Arye E, Schiff E, Vintal H, Agour O, Preis L, Steiner M. Integrating complementary medicine and supportive care: patients’ perspectives toward complementary medicine and spirituality. J Altern Complement Med. 2012;18:824–31. 10.1089/acm.2011.0327 22924415 10.1089/acm.2011.0327

[17] Heller T, Kloos C, Mueller N, Roemelt J, Keinki C, Wolf G, et al. Complementary and alternative medicine is positively associated with religiousness/spirituality. J Complement Integr Med. 2020;18:185–92. 10.1515/jcim-2018-0023 32562532 10.1515/jcim-2018-0023

[18] Institut für Demoskopie Allensbach. Naturheilmittel 2010 - Ergebnisse einer bevölkerungsrepräsentativen Befragung. 2010.

[19] Keene MR, Heslop IM, Sabesan SS, Glass BD. Complementary and alternative medicine use in cancer: A systematic review. Complement Ther Clin Pract. 2019;35:33–47. 10.1016/j.ctcp.2019.01.004 31003679 10.1016/j.ctcp.2019.01.004

[20] Bishop FL, Yardley L, Lewith GT. Treat or treatment: a qualitative study analyzing patients’ use of complementary and alternative medicine. Am J Public Health. 2008;98:1700–5. 10.2105/AJPH.2007.110072 18172145 10.2105/AJPH.2007.110072 PMC2509600

[21] Sointu E. The search for wellbeing in alternative and complementary health practices. Sociol Health Illn. 2006;28:330–49. 10.1111/j.1467-9566.2006.00495.x 16573719 10.1111/j.1467-9566.2006.00495.x

[22] Siahpush M. Postmodern values, dissatisfaction with conventional medicine and popularity of alternative therapies. J Sociol. 1998;34:58–70. 10.1177/144078339803400106

[23] Lamberty P, Imhoff R. Powerful pharma and its marginalized alternatives?? Social Psychol. 2018;49:255–70. 10.1027/1864-9335/a000347

[24] Oliver JE, Wood T. Medical conspiracy theories and health behaviors in the united States. JAMA Intern Med. 2014;174:817–8. 10.1001/jamainternmed.2014.190 24638266 10.1001/jamainternmed.2014.190

[25] eurostat. One in two EU citizens look for health information online. 6.4.2021. https://ec.europa.eu/eurostat/de/web/products-eurostat-news/-/edn-20210406-1. Accessed 8 Dec 2023.

[26] Swire-Thompson B, Lazer D. Public health and online misinformation: challenges and recommendations. Annu Rev Public Health. 2020;41:433–51. 10.1146/annurev-publhealth-040119-094127 31874069 10.1146/annurev-publhealth-040119-094127

[27] Dobransky K, Hargittai E. Inquiring Minds acquiring wellness: uses of online and offline sources for health information. Health Commun. 2012;27:331–43. 10.1080/10410236.2011.585451 21932982 10.1080/10410236.2011.585451

[28] Cotten SR, Gupta SS. Characteristics of online and offline health information seekers and factors that discriminate between them. Soc Sci Med. 2004;59:1795–806. 10.1016/j.socscimed.2004.02.020 15312915 10.1016/j.socscimed.2004.02.020

[29] Ayers SL, Kronenfeld JJ. Chronic illness and health-seeking information on the internet. Health (London). 2007;11:327–47. 10.1177/1363459307077547 17606698 10.1177/1363459307077547

[30] Slauson-Blevins KS, McQuillan J, Greil AL. Online and in-person health-seeking for infertility. Soc Sci Med. 2013;99:110–5. 10.1016/j.socscimed.2013.10.019 24355477 10.1016/j.socscimed.2013.10.019

[31] Bowes P, Stevenson F, Ahluwalia S, Murray E. I need her to be a Doctor’: patients’ experiences of presenting health information from the internet in GP consultations. Br J Gen Pract. 2012;62:e732–8. 10.3399/bjgp12X658250 23211176 10.3399/bjgp12X658250 PMC3481513

[32] Gilson L. Trust and the development of health care as a social institution. Soc Sci Med. 2003;56:1453–68. 10.1016/s0277-9536(02)00142-9 12614697 10.1016/s0277-9536(02)00142-9

[33] Birkhäuer J, Gaab J, Kossowsky J, Hasler S, Krummenacher P, Werner C, Gerger H. Trust in the health care professional and health outcome: A meta-analysis. PLoS ONE. 2017;12:e0170988. 10.1371/journal.pone.0170988 28170443 10.1371/journal.pone.0170988 PMC5295692

[34] Zhao Y, Zhang J. Consumer health information seeking in social media: a literature review. Health Info Libr J. 2017;34:268–83. 10.1111/hir.12192 29045011 10.1111/hir.12192

[35] Ramírez AS, Freres D, Martinez LS, Lewis N, Bourgoin A, Kelly BJ, et al. Information seeking from media and family/friends increases the likelihood of engaging in healthy lifestyle behaviors. J Health Commun. 2013;18:527–42. 10.1080/10810730.2012.743632 23472825 10.1080/10810730.2012.743632 PMC4254799

[36] Gray NJ, Klein JD, Noyce PR, Sesselberg TS, Cantrill JA. Health information-seeking behaviour in adolescence: the place of the internet. Soc Sci Med. 2005;60:1467–78. 10.1016/j.socscimed.2004.08.010 15652680 10.1016/j.socscimed.2004.08.010

[37] Dutta-Bergman MJ. Primary sources of health information: comparisons in the domain of health attitudes, health cognitions, and health behaviors. Health Commun. 2004;163:273–88. 10.1207/S15327027HC1603_1 10.1207/S15327027HC1603_1 15265751

[38] Finnegan JR, Viswanath K. Communication theory and health behavior change: the media studies framework. In: Glantz K, et al. editors. Health behavior and health education: theory, research and practice. 4ed ed. San Francisco: Jossey- Bass. 2008.

[39] Rosenstock IM. The health belief model and preventive health behavior. Health Educ Monogr. 1974;24:354–86. 10.1177/109019817400200405

[40] Rosenstock IM, Strecher VJ, Becker MH. Social learning theory and the health belief model. Health Educ Q. 1988;15(2):175–83. 3378902 10.1177/109019818801500203

[41] Jones CL, Jensen JD, Scherr CL, Brown NR, Christy K, Weaver J. The health belief model as an explanatory framework in communication research: exploring parallel, serial, and moderated mediation. Health Commun. 2015;30(6):566–76. Epub 2014 Jul 10. PMID: 25010519; PMCID: PMC4530978. 25010519 10.1080/10410236.2013.873363 PMC4530978

[42] Mood C. Logistic regression: why we cannot do what we think we can do, and what we can do about it. Eur Sociol Rev. 2010;26:67–82. 10.1093/esr/jcp006

[43] Pearson SD, Raeke LH. Patients’ trust in physicians: many theories, few measures, and little data. J GEN INTERN MED. 2000;15:509–13. 10.1046/j.1525-1497.2000.11002.x 10940139 10.1046/j.1525-1497.2000.11002.x PMC1495476

[44] Robinson A, McGrail MR. Disclosure of CAM use to medical practitioners: a review of qualitative and quantitative studies. Complement Ther Med. 2004;12:90–8. 10.1016/j.ctim.2004.09.006 15561518 10.1016/j.ctim.2004.09.006

[45] Meppelink CS, Smit EG, Fransen ML, Diviani N. I was right about vaccination: confirmation Bias and health literacy in online health information seeking. J Health Commun. 2019;24:129–40. 10.1080/10810730.2019.1583701 30895889 10.1080/10810730.2019.1583701

[46] Schweiger S, Cress U. How confidence in prior attitudes, social Tag popularity, and source credibility shape confirmation Bias toward antidepressants and psychotherapy in a representative German sample: randomized controlled Web-Based study. J Med Internet Res. 2019;21:e11081. 10.2196/11081 31012865 10.2196/11081 PMC6658248

[47] Moreno-Fernández MM, Matute H. Biased sampling and causal Estimation of Health-Related information: Laboratory-Based experimental research. J Med Internet Res. 2020;22:e17502. 10.2196/17502 32706735 10.2196/17502 PMC7414405

