# Supplementary material for: Health information-seeking behavior among users of traditional, complementary and integrative medicine (TCIM)
Source: BMC Complement Med Ther. 2025 Mar 21;25:111. doi: 10.1186/s12906-025-04843-9 (PMC11927221; doi:10.1186/s12906-025-04843-9)
Supplement: Supplementary file 1 — Supplementary Material 1 [file 12906_2025_4843_MOESM1_ESM.docx]

**Online Supplement**

By means of PCA we identified two dimensions based on the Eigenvalue criterion (>1) which explain 61.4 percent of the variance. The Kaiser-Meyer-Olkin measure yielded 0.79, indicating that sampling was adequate. Interpretations of the two components are based on high loadings after varimax rotation (>0.3). The first latent motive, *health-promoting measure*, is based on the high loadings for the first four items (retrieved factor loading range from -5.1 to 2.6). The second component has high loadings for the last three items, combined to represent *aversion towards conventional medicine* (retrieved factor loading range from -2.6 to 3.5). Details on the PCA are shown in table S1 and table S2. Multivariate analyses based on the retrieved scores by the PCA (table S3 and table S4) yield almost identical results compared to the multivariate analysis based on the indices (see main paper table A2 and A3).

Table S1: Principal component analysis of the items of motives for TCIM use: Total variance explained (N=1,366)

|  | Total Variance | Percent of Variance | Cumulative Percent |
| --- | --- | --- | --- |
| Component 1 | 2.4 | 34.4 | 34.4 |
| Component 2 | 1.9 | 26.9 | 61.3 |

Note: Authors’ own calculations.

Table S2: Principal component analysis of the items of working atmosphere: rotated component matrix (N=1,366)

|  | Dimensions | |  |
| --- | --- | --- | --- |
| Items | 1 | 2 |  |
| Fewer side effects than with conventional medicine | **0.54** | -0.05 | Health-promoting measure |
| Reduction of side effects of conventional medication | **0.54** | -0.04 |  |
| Better chances of recovery | **0.42** | 0.11 |  |
| Improving health literacy and self-care competencies | **0.47** | 0.02 |  |
| I do it out of health-related desperation | -0.02 | **0.53** | aversion towards conventional medicine |
| I don’t like conventional medicine | -0.06 | **0.63** |  |
| I have had bad experiences with conventional medicine | 0.08 | **0.55** |  |

Note: Rotation method: varimax, loadings larger than 0.3 are in bold. Authors’ own calculations.

Table S3: Multinomial regression of most important medical information source on reason for using TCIM, based on indices (AMEs/ADCs)

|  | M1b | | | | | |
| --- | --- | --- | --- | --- | --- | --- |
|  | medical professionals | | (online) media outlet | | social circle | |
|  | AME/ADC  (Std. err.) | | AME/ADC  (Std. err.) | | AME/ADC  (Std. err.) | |
| health-promoting measure | -0.01 | (0.01) | 0.01 | (0.01) | -0.00 | (0.00) |
| aversion towards conventional medicine | -0.04^***^ | (0.01) | 0.04^***^ | (0.01) | 0.01 | (0.00) |
| Confounders: |  |  |  |  |  |  |
| gender (ref.: man) |  |  |  |  |  |  |
| woman | 0.03 | (0.03) | -0.03 | (0.03) | 0.01 | (0.01) |
| *diverse* | 0.05 | (0.28) | -0.00 | (0.28) | -0.05^***^ | (0.01) |
| age | 0.00 | (0.00) | -0.00 | (0.00) | -0.00 | (0.00) |
| hometown size | -0.02^***^ | (0.01) | 0.02^**^ | (0.01) | 0.01 | (0.00) |
| net equivalent income | 0.00^***^ | (0.00) | -0.00^**^ | (0.00) | -0.00 | (0.00) |
| level of education (ref.: low) |  |  |  |  |  |  |
| medium | -0.03 | (0.03) | 0.00 | (0.03) | 0.02 | (0.02) |
| high | -0.02 | (0.03) | -0.01 | (0.03) | 0.02 | (0.02) |
| *currently enrolled* | -0.05 | (0.19) | 0.08 | (0.19) | -0.03^**^ | (0.01) |
| work status (ref.: full time) |  |  |  |  |  |  |
| part time | 0.03 | (0.04) | -0.04 | (0.04) | 0.01 | (0.02) |
| in training/school | 0.17^***^ | (0.04) | -0.15^***^ | (0.04) | -0.02 | (0.02) |
| not working | 0.00 | (0.04) | -0.02 | (0.04) | 0.02 | (0.02) |
| previous medical training (ref.: no) | -0.06 | (0.04) | 0.06 | (0.04) | 0.00 | (0.02) |
| subjective health status | -0.05^**^ | (0.02) | 0.04^*^ | (0.01) | 0.01 | (0.01) |
| level of spirituality | -0.02 | (0.01) | 0.01 | (0.01) | 0.01 | (0.00) |
| importance of digitality | -0.01 | (0.01) | 0.02 | (0.01) | -0.01 | (0.01) |
| important initial source of influence for TCIM use (ref.: no) |  |  |  |  |  |  |
| good exp. of family/friends | 0.01 | (0.03) | -0.03 | (0.03) | 0.01 | (0.01) |
| advice from treating physician | 0.13^***^ | (0.03) | -0.12^***^ | (0.02) | -0.01 | (0.01) |
| info in the media | -0.08^*^ | (0.03) | 0.07^*^ | (0.03) | 0.01 | (0.01) |
| N | 1,366 | | | | | |

Note: Significance level: * p < 0.05, ** p < 0.01, *** p < 0.001*.* Items displayed in italics should not be interpreted due to low cell size. Authors’ own calculations.

Table S4: Logistic regression of most important medical information source on reason for using TCIM, based on indices (AMEs/ADCs)

|  | M2b | | M3b | | M4b | | M5b | |
| --- | --- | --- | --- | --- | --- | --- | --- | --- |
|  | Scientific studies | | Advice by doctor | | Personal advice | | Experience by social circle | |
|  | AME/ADC  (Std. err.) | | AME/ADC  (Std. err.) | | AME/ADC  (Std. err.) | | AME/ADC  (Std. err.) | |
| health-promoting measure | 0.03^***^ | (0.01) | 0.01 | (0.01) | 0.05^***^ | (0.01) | 0.05^***^ | (0.01) |
| aversion towards conv. medicine | -0.03^**^ | (0.01) | -0.05^***^ | (0.01) | 0.01 | (0.01) | 0.00 | (0.01) |
| Confounders: |  |  |  |  |  |  |  |  |
| gender (ref.: man) |  |  |  |  |  |  |  |  |
| woman | 0.00 | (0.03) | -0.05^*^ | (0.02) | 0.01 | (0.03) | 0.03 | (0.03) |
| *diverse* | - | - | -0.37 | (0.27) | -0.00 | (0.28) | -0.09 | (0.29) |
| age | 0.00 | (0.00) | 0.00^***^ | (0.00) | 0.00 | (0.00) | 0.00 | (0.00) |
| hometown size | 0.00 | (0.01) | -0.00 | (0.01) | 0.01 | (0.01) | 0.02^*^ | (0.01) |
| net equiv. income | 0.00 | (0.00) | 0.00 | (0.00) | 0.00^*^ | (0.00) | 0.00 | (0.00) |
| level of education (ref.: low) |  |  |  |  |  |  |  |  |
| medium | 0.06 | (0.04) | -0.00 | (0.03) | -0.01 | (0.03) | -0.10^**^ | (0.03) |
| high | 0.15^***^ | (0.04) | 0.04 | (0.03) | -0.07 | (0.03) | -0.11^***^ | (0.03) |
| *curr. enrolled* | 0.01 | (0.19) | -0.06 | (0.15) | -0.26 | (0.21) | - | - |
| work status (ref.: full time) |  |  |  |  |  |  |  |  |
| part time | 0.07 | (0.04) | 0.03 | (0.03) | 0.02 | (0.04) | 0.00 | (0.04) |
| in training/school | 0.11^*^ | (0.05) | 0.08^*^ | (0.04) | 0.05 | (0.05) | 0.05 | (0.05) |
| not working | 0.08^*^ | (0.04) | 0.03 | (0.03) | -0.04 | (0.04) | -0.05 | (0.04) |
| previous medical training (ref.: no) | -0.04 | (0.04) | -0.03 | (0.03) | 0.02 | (0.04) | -0.00 | (0.03) |
| subj. health status | -0.04^*^ | (0.02) | -0.03^*^ | (0.01) | 0.02 | (0.02) | 0.02 | (0.02) |
| level of spirituality | -0.02 | (0.01) | -0.03^***^ | (0.01) | 0.03^**^ | (0.01) | 0.01 | (0.01) |
| importance of digitality | 0.06^***^ | (0.01) | 0.03^***^ | (0.01) | -0.00 | (0.01) | -0.02 | (0.01) |
| important initial source of influence for TCIM use (ref.: no) |  |  |  |  |  |  |  |  |
| good exp. of family/friends | 0.02 | (0.03) | 0.06^*^ | (0.02) | 0.24^***^ | (0.02) | 0.29^***^ | (0.02) |
| advice from treating physician | 0.14^***^ | (0.02) | 0.25^***^ | (0.02) | 0.02 | (0.03) | 0.00 | (0.03) |
| info in the media | 0.16^***^ | (0.03) | 0.01 | (0.03) | 0.05 | (0.03) | -0.02 | (0.03) |
| N | 1,363 | | 1,366 | | 1,366 | | 1,360 | |

Note: Significance level: * p < 0.05, ** p < 0.01, *** p < 0.001*.* Items displayed in italics should not be interpreted due to their low cell size. In Model 2b and 5b some observations were omitted as there is no variation in the dependent variable for people who are diverse/currently enrolled. Authors’ own calculations.
