# Supplementary material for: Health information-seeking behavior among users of traditional, complementary and integrative medicine (TCIM)
Source: BMC Complement Med Ther. 2025 Mar 21;25:111. doi: 10.1186/s12906-025-04843-9 (PMC11927221; doi:10.1186/s12906-025-04843-9)
Supplement: Supplementary file 2 — Supplementary Material 2 [file 12906_2025_4843_MOESM2_ESM.docx]

**Appendix**

Table A1: Descriptive sample characteristics, in percent (N=1,696)

| Gender |  |
| --- | --- |
| men | 39.8 |
| women | 59.9 |
| diverse | 0.3 |
| Age, mean (SD) | 50 (16) |
| Hometown size |  |
| under 2000 inhab. | 7.0 |
| 2,000 to under 5,000 inhab. | 5.8 |
| 5,000 to under 20,000 inhab. | 15.2 |
| 20,000 to under 50,000 inhab. | 14.7 |
| 50,000 to under 100,000 inhab. | 9.7 |
| 100,000 to under 500,000 inhab. | 21.5 |
| 500,000 inhab. and more | 26.1 |
| Net equivalent income, mean (SD) | 1785 (896) |
| Educational level |  |
| in school | 0.4 |
| low | 25.9 |
| medium | 29.6 |
| high | 44.1 |
| Work status |  |
| full time | 38.9 |
| part time | 16.1 |
| in training/school | 6.9 |
| not working | 38.1 |
| Medical training |  |
| yes | 12.9 |
| no | 87.2 |
| Subjective health status |  |
| bad | 4.1 |
| less good | 24.2 |
| good | 47.5 |
| very good | 21.8 |

|  |  |
| --- | --- |

Table A1 (continued)

| Level of spirituality |  |
| --- | --- |
| not at all | 28.2 |
| not really | 22.1 |
| neither nor | 15.9 |
| yes, somewhat | 25.0 |
| yes, very | 6.6 |
| don’t know | 2.2 |
| Digital technology plays central role in my life |  |
| does not apply at all | 6.6 |
| does not really apply | 14.0 |
| neutral | 29.0 |
| applies somewhat | 37.1 |
| fully applies | 12.4 |
| don’t know | 0.9 |
| important initial source of influence for TCIM use |  |
| good experiences of family/friends | 46.2 |
| advice from treating physician | 63.6 |
| information in the media | 19.7 |

Note: Authors’ own calculations, unweighted

Table A2: Multinomial regression of most important medical information source on reason for using TCIM, based on indices

|  | M1b | | | | | |
| --- | --- | --- | --- | --- | --- | --- |
|  | medical profess. | | media outlet | | social circle | |
|  | AME/ADC  (Std. err.) | | AME/ADC  (Std. err.) | | AME/ADC  (Std. err.) | |
| health-promoting measure | -0.01 | (0.02) | -0.02 | (0.01) | -0.01 | (0.01) |
| aversion towards conventional medicine | -0.06^***^ | (0.01) | 0.05^***^ | (0.01) | 0.01 | (0.01) |
| Confounders: |  |  |  |  |  |  |
| gender (ref.: man) |  |  |  |  |  |  |
| woman | 0.01 | (0.02) | -0.02 | (0.02) | 0.01 | (0.01) |
| *diverse* | 0.10 | (0.21) | -0.05 | (0.21) | -0.05^***^ | (0.01) |
| age | 0.00^**^ | (0.00) | -0.00 | (0.00) | -0.00 | (0.00) |
| hometown size | -0.02^***^ | (0.01) | 0.01^*^ | (0.01) | 0.01^*^ | (0.00) |
| net equivalent income | 0.00^**^ | (0.00) | -0.00^**^ | (0.00) | -0.00 | (0.00) |
| level of education (ref.: low) |  |  |  |  |  |  |
| medium | -0.03 | (0.03) | 0.03 | (0.03) | 0.01 | (0.01) |
| high | -0.01 | (0.03) | 0.01 | (0.03) | 0.01 | (0.01) |
| *currently enrolled* | -0.16 | (0.18) | 0.04 | (0.17) | 0.12 | (0.15) |
| work status (ref.: full time) |  |  |  |  |  |  |
| part time | 0.02 | (0.03) | -0.02 | (0.03) | -0.00 | (0.02) |
| in training/school | 0.18^***^ | (0.04) | -0.16^***^ | (0.04) | -0.03 | (0.02) |
| not working | 0.03 | (0.03) | -0.02 | (0.03) | -0.00 | (0.02) |
| former medical training (ref.: no) | -0.07^*^ | (0.03) | 0.06^*^ | (0.03) | 0.00 | (0.02) |
| subjective health status | -0.05^**^ | (0.01) | 0.04^**^ | (0.01) | 0.01 | (0.01) |
| level of spirituality | -0.02^**^ | (0.01) | 0.01 | (0.01) | 0.01 | (0.00) |
| importance of digitality | -0.01 | (0.01) | 0.02^*^ | (0.01) | -0.01^**^ | (0.01) |
| important initial source of influence for TCIM use (ref.: no) |  |  |  |  |  |  |
| good exp. of family/friends | 0.02 | (0.02) | -0.04 | (0.02) | 0.02 | (0.01) |
| advice from doctor | 0.14^***^ | (0.02) | -0.14^***^ | (0.02) | -0.01 | (0.01) |
| info in the media | -0.08^**^ | (0.03) | 0.08^**^ | (0.03) | 0.00 | (0.01) |
| N | 1,696 | | | | | |

Note: Significance level: * p < 0.05, ** p < 0.01, *** p < 0.001*.* Items displayed in italics should not be interpreted due to low cell size. Authors’ own calculations.

Table A3: Logistic regression of most important medical information source on reason for using TCIM, based on indices

|  | M2b | | M3b | | M4b | | M5b | |
| --- | --- | --- | --- | --- | --- | --- | --- | --- |
|  | Scientific studies | | Advice by doctor | | Personal advice | | Experience by social circle | |
|  | AME/ADC  (Std. err.) | | AME/ADC  (Std. err.) | | AME/ADC  (Std. err.) | | AME/ADC  (Std. err.) | |
| health-promoting measure | 0.04^**^ | (0.01) | -0.00 | (0.01) | 0.09^***^ | (0.01) | 0.08^***^ | (0.01) |
| aversion towards conv. medicine | -0.04^**^ | (0.01) | -0.07^***^ | (0.01) | 0.02 | (0.01) | 0.01 | (0.01) |
| Confounders: |  |  |  |  |  |  |  |  |
| gender (ref.: man) |  |  |  |  |  |  |  |  |
| woman | 0.00 | (0.02) | -0.04^*^ | (0.02) | 0.01 | (0.02) | 0.03 | (0.02) |
| *diverse* | 0.23 | (0.16) | -0.01 | (0.18) | -0.27 | (0.16) | -0.18 | (0.20) |
| age | 0.00 | (0.00) | 0.00^***^ | (0.00) | 0.00 | (0.00) | -0.00 | (0.00) |
| hometown size | 0.00 | (0.01) | -0.00 | (0.01) | 0.01 | (0.01) | 0.01^**^ | (0.01) |
| net equiv. income | 0.00* | (0.00) | 0.00 | (0.00) | 0.00^*^ | (0.00) | 0.00 | (0.00) |
| level of education (ref.: low) |  |  |  |  |  |  |  |  |
| medium | 0.04 | (0.03) | 0.00 | (0.03) | -0.00 | (0.03) | -0.07^*^ | (0.03) |
| high | 0.14^***^ | (0.03) | 0.05 | (0.03) | -0.07^*^ | (0.03) | -0.10^***^ | (0.03) |
| *curr. enrolled* | -0.07 | (0.18) | -0.19 | (0.17) | -0.30 | (0.19) | - | - |
| work status (ref.: full time) |  |  |  |  |  |  |  |  |
| part time | 0.05 | (0.04) | 0.02 | (0.03) | 0.04 | (0.04) | 0.02 | (0.03) |
| in training/school | 0.11^*^ | (0.05) | 0.07^*^ | (0.03) | 0.04 | (0.05) | 0.04 | (0.05) |
| not working | 0.06 | (0.03) | 0.04 | (0.03) | -0.05 | (0.03) | -0.04 | (0.03) |
| former medical training (ref.: no) | -0.03 | (0.03) | -0.04 | (0.03) | 0.03 | (0.03) | -0.00 | (0.03) |
| subj. health status | -0.03^*^ | (0.01) | -0.03^*^ | (0.01) | 0.02 | (0.01) | 0.02 | (0.01) |
| level of spirituality | -0.01 | (0.01) | -0.03^***^ | (0.01) | 0.03^***^ | (0.01) | 0.00 | (0.01) |
| importance of digitality | 0.07^***^ | (0.01) | 0.03^***^ | (0.01) | -0.00 | (0.01) | -0.01 | (0.01) |
| important initial source of influence for TCIM use (ref.: no) |  |  |  |  |  |  |  |  |
| good exp. Of  family/friends | 0.03 | (0.02) | 0.06^**^ | (0.02) | 0.23^***^ | (0.02) | 0.31^***^ | (0.02) |
| advice from doctor | 0.13^***^ | (0.02) | 0.24^**^ | (0.02) | 0.02 | (0.02) | -0.00 | (0.02) |
| info in the media | 0.18^***^ | (0.03) | 0.03 | (0.02) | 0.04 | (0.03) | -0.03 | (0.03) |
| N | 1,696 | | 1,696 | | 1,696 | | 1,689 | |

Note: Significance level: * p < 0.05, ** p < 0.01, *** p < 0.001*.* Items displayed in italics should not be interpreted due to their low cell size. In Model 5b seven observations were omitted as there is no variation in the dependent variable for people currently enrolled. Authors’ own calculations.
